# Nanopore sequencing for the screening of myeloid and lymphoid neoplasms with eosinophilia and rearrangement of PDGFRα, PDGFRβ, FGFR1 or PCM1-JAK2

**DOI:** 10.1186/s40364-021-00337-1

**Published:** 2021-11-12

**Authors:** Simone Romagnoli, Niccolò Bartalucci, Francesca Gesullo, Manjola Balliu, Stefania Bonifacio, Anair Graciela Lema Fernandez, Francesco Mannelli, Davide Bolognini, Elisabetta Pelo, Cristina Mecucci, Paola Guglielmelli, Alessandro Maria Vannucchi

**Affiliations:** 1 CRIMM, Center of Research and Innovation of Myeloproliferative Neoplasms, Department of Experimental and Clinical medicine, Careggi University Hospital, University of Florence, DENOTHE Excellence Center, CUBO 3, Padiglione 27b, Viale Pieraccini, 6, 50134 Florence, Italy; 2 Genetic Diagnostic Unit, Careggi University Hospital, Florence, Italy; 3 Hematology Unit, Laboratory of Cytogenetics and Molecular Medicine, University of Perugia, Perugia, Italy; 4 Unit of Medical Genetics, Meyer Children’s Hospital, Florence, Italy

**Keywords:** Primary eosinophilic disorders, Nanopore sequencing, *PDGFRα*, *PDGFRβ*, *FGFR1*

## Abstract

Eosinophilia represents a group of diseases with heterogeneous pathobiology and clinical phenotypes. Among the alterations found in primary Eosinophilia, gene fusions involving *PDGFRα*, *PDGFRβ*, *FGFR1* or *JAK2* represent the biomarkers of WHO-defined “myeloid and lymphoid neoplasms with eosinophilia”. The heterogeneous nature of genomic aberrations and the promiscuity of fusion partners, may limit the diagnostic accuracy of current cytogenetics approaches. To address such technical challenges, we exploited a nanopore-based sequencing assay to screen patients with primary Eosinophilia. The comprehensive sequencing approach described here enables the identification of genomic fusion in 60 h, starting from DNA purified from whole blood.

**Supplementary Information:**

The online version contains supplementary material available at 10.1186/s40364-021-00337-1.

To the Editor,

the 2016-WHO category of myeloid and lymphoid neoplasms with eosinophilia and abnormalities of *PDGFRα*, *PDGFRβ*, *FGFR1* or *PCM1-JAK2* (MLN-Eo) is defined by an absolute, persistent, eosinophil count (AEC) ≥1500/uL [1]. In most cases, the initial diagnostic framework relies on cytogenetics; individual molecular probes specifically targeting *PDGFRα*, *PDGFRβ*, *FGFR1* or the *PCM1-JAK2* fusion are employed for FISH analysis to identify the most recurrent translocations. However, owing to the promiscuous nature of the fusion events [2], including currently unknown partners, FISH approach has anticipated shortcomings depending on the availability of probes for known partner genes [3]. On the other hand, RNA analysis might be more informative but it poses long turnaround times and bioinformatic challenges [4]. In this context, we exploited the potential advantages of a long-read genome-wide nanopore sequencing (NS) to detect fusion events involving *PDGFRα/β, FGFR1 and JAK2* in unamplified DNA samples [5].

To the purposes of the study, we sequenced 12 samples from patients with Eosinophilia (7 males, 5 females) whose familiar or secondary origin were excluded and who had stored samples collected at presentation (local ethics committee approval: #14,560). Full set of clinical and cytogenetic data were available for all the patients (Supplemental Table 1). The median age, AEC and white blood cell count at diagnosis were, respectively, 48 years (range 25-85), 1.4/L (range, 1.1-6.7) and 14.45 × 10^9^/L (range, 7.3-105). All subjects were negative for *JAK2*^*V617F*^, *MPL*^*W515*^ or *CALR*^*exon9*^ mutation.

Genomic DNA was purified from whole blood and prepared for whole genome NS as previously described [6]. Rough sequencing data were aligned to the Human Reference GRCh38 by Minimap2 (v2.17). Variant calling in the regions of interest was carried out through a read-count approach (Fig. 1 A) with Nano-GLADIATOR [7], and by a gapped-alignment and split-read approach (Fig. 1 B) through Sniffles [8].

**Fig. 1:**
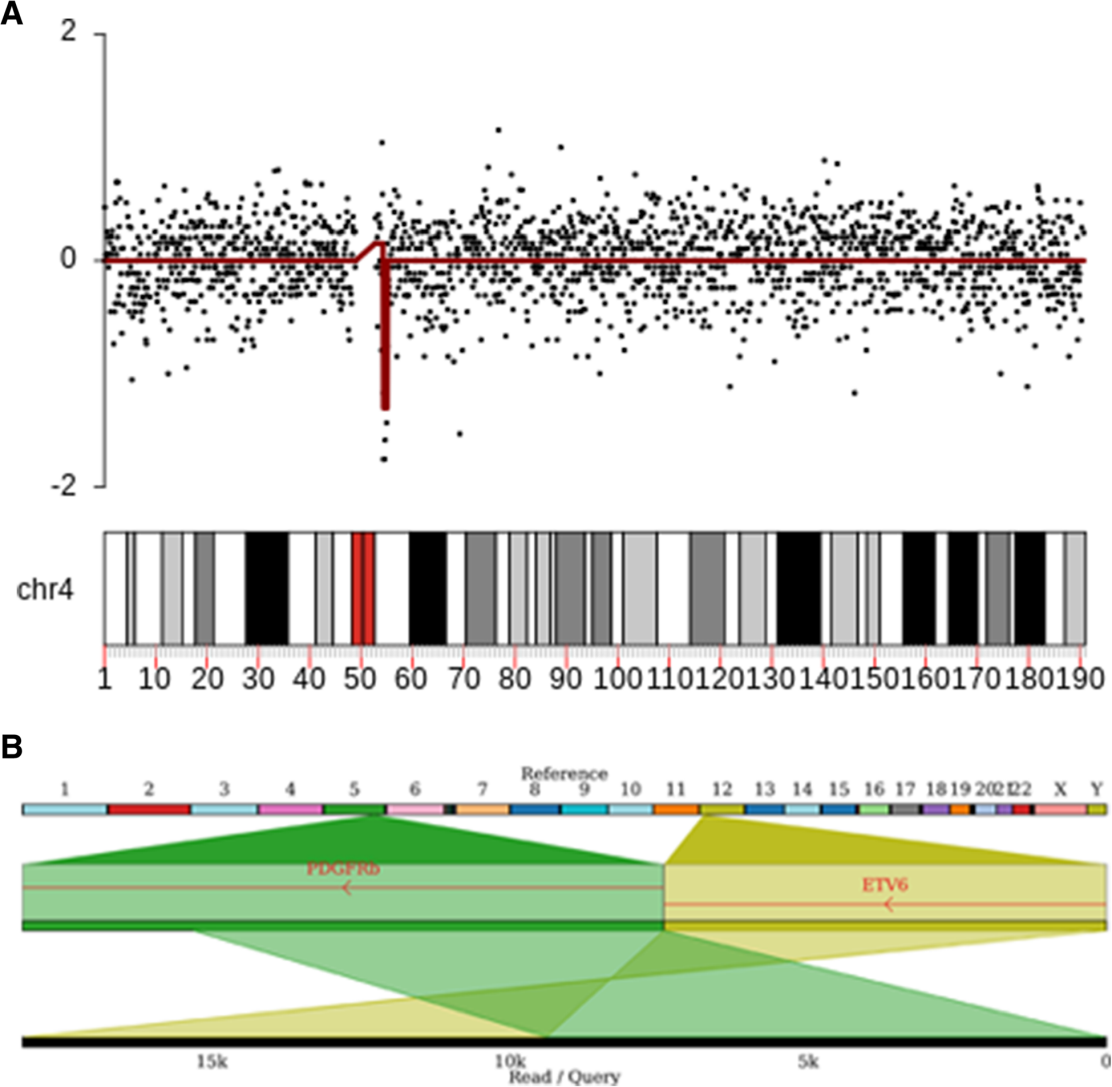
Visualization of genomic variants in two representative samples. Panel **A** shows the interstitial deletion at chr4(q12) detected in sample #1 and visualized by KaryoploteR. In the chart, the log2 copy ratio values, on the Y axis, reflects the ploidy along the chromosome. The black dots represent log2 values for each examined window (log2 ratio=0 for diploid region); the copy number segmentation of the log2 ratio is visualized by the red line. Segments were assigned gain, loss or normal copy basing on cut-off estimated by the within-segment standard deviation of post-normalized signals. The signal reduction point at the loss of genomic material caused by the del[4](q12q12). Panel **B** shows a chimeric read isolated in sample #4 resulting from the fusion between chromosome 5 (green) and chromosome 12 (dark yellow), visualized by Ribbon. The chimeric read spanning 18,108 bp, of which 8,756 bp mapped on chromosome 12 and 9,352 bp on chromosome 5, represents the molecular marker of the t(5;12)(q33;p13) detected in the sample

Given the prevalence of the translocation *FIP1L1-PDGFRα* in MLN-Eo, we first performed a read-count analysis aimed at detecting possible interstitial deletion involving *PDGFRα* [9]. A del [4](q12q12) was identified in 3 samples, involving 800±100Kb (sample #1), 700±100Kb (sample #2) and 900±100Kb (sample #3). Further annotation by AnnotSV [10] revealed the genes comprised by the reported deletions, as shown in Table 1.

**Table 1:** Genomic variants detected in the samples cohort. The table summarizes the nanopore sequencing and the F.I.S.H. results for each patient included in the study. The genomic coordinates of the fusion breakpoint and the genes involved by the alteration are provided for each variant reported. No fusion event was detected (ND, Not Detected) in the patients indicated as normal karyotype (46, XX or XY) by FISH and NS analysis

Sample	F.I.S.H.	NS		
	*karyotype*	*karyotype*	*Involved Genes*	*Fusion Breakpoint*
#1	46, XX,del(4)(q12q12)	46, XX,del(4)(q12q12)	*LNX1, LNX1-AS2, LOC100506444, RPL21P44, CHIC2, GSX2, PDGFRα*	Chr4:53,443,951 - Chr4:54,343,951
#2	46, XY,del(4)(q12q12)	46, XY,del(4)(q12q12)	*LNX1*, *LNX-AS2*, *RPL21P44*, *CHIC2, GSX2*	Chr4:53,543,951 – Chr4:54,343,951
#3	46, XY,del(4)(q12q12)	46, XY,del(4)(q12q12)	*FIP1L1 (16Kb), LNX1, LNX1-AS1, LNX1-AS2, LOC100506444, RPL21P44, CHIC2, GSX2, PDGFRα, LINC0228*	Chr4:53,443,951 – Chr4:54,143,951
#4	46, XY,t(5;12)(q32;p13)	46, XY,t(5;12)(q32;p13)	*PDGFRβ-ETV6*	Chr5:150,129,614 – Chr12:11,867,739
#5	46, XY,t(5;14)(q32q32)	46, XY,t(5;14)(q32q32)	*PDGFRβ-CCDC88C*	Chr5:150,129,617 – Chr14:91,290,817
#6	46, XX,t(8;13)(p11;q12)	46, XX,t(8;13)(p11;q12)	*FGFR1-ZMYM2*	Chr8:38,417,891 – Chr13:20,059,507
#7	46, XX, t(8;13)(p11;q12)	46, XX, t(8;13)(p11;q12)	*FGFR1-ZMYM2*	Chr8:38,957,173 – Chr13:206,235,585
#8	46, XX	46, XX	ND	ND
#9	46, XY	46, XY	ND	ND
#10	46, XY	46, XY	ND	ND
#11	46, XY	46, XY	ND	ND
#12	46, XX	46, XX	ND	ND

Sequencing data were further analysed by Sniffles. In samples #4 and #5, chimeric reads with multiple alignment pointing were detected at a t(5;12)(q32;p13) and a t(5;14)(q32q32), respectively. The chimeric reads in sample #4 spanned from 9,394 bp to 52,545 bp, of which at least 810 bp (up to 46,423 bp) were aligned to *PDGFRβ* and 6,108 bp (up to 21,245 bp) to *ETV6*; more specifically, the clustering of chimeric reads predicted the fusion breakpoint between intron 10 of *PDGFRβ* (nucleotide, *nt*, position 15,776) and intron 4 of *ETV6* (*nt* position 218,066). The translocation found in sample #5 was originated by the fusion between *PDGFRβ* intron 9 (*nt* position 16,372) and *CCDC88C* intron 24 (*nt* position 19,495). The chimeric read spanned 32,847 bp, where 22,736 bp were aligned to *PDGFRβ* and 9,111 bp to *CCDC88C*.

In samples #6 and #7 we found, respectively, 3 and 2 chimeric reads predicting for a t(8;13)(p11;q12). The chimeric reads in sample #6 (spanning from 16,164 bp to 15,152 bp) were composed by the *FGFR1* sequence (min overlap: 678 bp – max overlap: 7,324 bp) fused to *ZMYM2* (min overlap: 8,911 bp – max overlap: 25,858 bp); the fusion breakpoint was located at nucleotides 6,754 and 102,102 of *FGFR1* and *ZMYM2*, respectively. In sample #7, the two chimeric reads (22,890 bp and 57,953 bp) were aligned to *FGFR1* (by 13,483 or 21,753 bp) and to *ZMYM2* (by 9,407 or 36,200 bp). The fusion breakpoint was detected between nucleotides 21,209 of *FGFR1* and 89,374 of *ZMYM2*.

No *PCM1-JAK2* fusion was detected in any samples of the cohort.

The NS screening results were in full agreement with FISH analysis (Pearson’s R^2^ coefficient:1) independently performed on the same samples of eosinophils collected at diagnosis. We show here that long-reads analysis facilitated the identification of the exact breakpoints of gene fusion in the 7 mutated patients, an information not provided by conventional cytogenetic approaches. The described pipeline allows to complete simultaneous genomic search for rearrangements of *PDGFRα/β, FGFR1* and *JAK2* in 60 h from blood sample collection, at an affordable cost, currently estimated at 500 Euros per sample. Finally, the NS long-reads sequencing of DNA enables the identification of possible unknown fusion partners by the alignment of the chimeric sequences to a reference genome.

## Supplementary information

**Additional file 1.**

## Data Availability

The datasets analyzed during the study are available at the Gene Expression Omnibus (GEO) database repository: GEO code GSE185446 (https://www.ncbi.nlm.nih.gov/geo/query/acc.cgi?acc=GSE185446*)*.

## Abbreviations

AEC: Absolute Eosinophil Count
MLN-Eo: myeloid and lymphoid neoplasms with eosinophilia and abnormalities of *PDGFRα*, *PDGFRβ*, *FGFR1* or *PCM1-JAK2*
FISH: Fluorescence In Situ Hybridization
NS: Nanopore Sequencing
nt: nucleotide
