# Supplementary material for: Nanopore sequencing for the screening of myeloid and lymphoid neoplasms with eosinophilia and rearrangement of PDGFRα, PDGFRβ, FGFR1 or PCM1-JAK2
Source: Biomark Res. 2021 Nov 12;9:83. doi: 10.1186/s40364-021-00337-1 (PMC8588648; doi:10.1186/s40364-021-00337-1)
Supplement: Supplementary file 1 — Additional file 1. [file 40364_2021_337_MOESM1_ESM.docx]

**Supplemental Information**

**Supplemental Table I. Baseline information of study cohort**

| **Clinical parameter** | **Value** |
| --- | --- |
| Age at diagnosis – *median (min-max)* | 48 (25-85) yr |
| Sex – *male:female* | 7:5 |
| Neutrophils – *mean (min-max)* | 52 (34-82) % |
| Eosinophils – *mean (min-max)* | 16.9 (4-44) % |
| Basophils – *mean (min-max)* | 1.4 (0-6) % |
| Leukocyte – *mean (min-max)* | 17 (4-55) % |
| Monocyte - *mean (min-max)* | 7.7 (0-28) % |
| AEC – *median (min-max)* | 1.4 (1.1-6.7) /L |
| WBC – *median (min-max)* | 14.45 (7.3 – 105) x10^9^/L |
| RBC – *median (min-max)* | 3.38 (3.19-6.16) x10^12^/L |
| Hb – *median (min-max)* | 12 (8.4-13.9) g/dL |
| Hct – *median (min-max)* | 33.7 (26.5-44.7) % |
| Plts – *median (min-max)* | 134 (25-277) x10^9^/L |
| LDH – *median (min-max)* | 329 (155-433) U/L |
